# First report of root-lesion nematode, *Pratylenchus oleae* from *p*istachio in Iran

**DOI:** 10.21307/jofnem-2021-103

**Published:** 2021-12-08

**Authors:** Farhad Saeidi Naeini, Zahra Majd Taheri

**Affiliations:** 1 Nematology Department, Iranian Research Institute of Plant Protection, Agricultural Research, Education and Extension, Organization (AREEO), Tehran, Iran

**Keywords:** Molecular taxonomy, Phylogeny, *Pistacia vera*, Pratylenchus

## Abstract

Pistachio, *Pistacia vera* is one of the most important cash crops in Iran that is scattered in arid and semi-arid regions. During a survey of plant-parasitic nematodes of pistachio in Ardakan city in Yazd Province, a species of root-lesion nematode was isolated and identified by morphological, morphometrical, and molecular methods as *Pratylenchus oleae* Palomares-Rius et al., 2014. This species was isolated from several pistachio trees rhizosphere regarding to molecular analysis, D2–D3 expansion segments of 28S rRNA was amplified by PCR and sequenced. The sequence was deposited in GenBank (Accession No. MW338666). Along with the related phylogenetic analysis, placed this species in a monophyletic clade with other *Pratylenchus oleae* isolates, based on Bayesian inference (BI) phylogeny. A PCR reaction with the *P. oleae* specific primer set produced a 547 bp fragment. This is the first report of *P. oleae* infecting Pistachio tree in the world.

Pistachio is a member of the Anacardiaceae family and belongs to the genus *Pistacia*. *P. vera* is one of the most important commercial cultivars among the 11 identified species. More than 498,000 hectares of Iranian lands are cultivated with pistachio trees and its product with nearly 173000 tones has a high domestic and foreign acceptance (Ahmadi et al., 2018). At present, Iran is the largest producer and exporter of this product in the world. Several plant parasitic nematode species were having been identified from pistachio rhizospheres. Plant endo-parasitic nematodes are more important in terms of quantitative and qualitative damage to plants and root-lesion nematodes are considered in terms of damage in this category. Root-lesion nematodes species are considered as the third most important group of plant-parasitic nematodes affecting worldwide crop. They are widely distributed in pistachio orchards and reduce yields (Castillo and Vovlas, 2007). The genus *Pratylenchus* Filipjev, 1936 includes approximately 100 valid species. Traditionally, identification of the species relies on morphology, morphometric and molecular methods (Gafur, 2020). So far, three root-lesion nematode species including *P. neglectus*, *P. pseudopratensis* and *P. thornei* have been isolated and reported from pistachio trees in Kerman, Qazvin, Qom, Yazd, and Fars provinces from Iran (Alvani et al., 2016). The aim of this work was to provide morphological, morphometrically and molecular characters of *P. oleae* from Iran.

## Materials and methods

### Nematode sampling and morphological identification

In the study of pistachio orchards throughout Iran during 2019–2020, several soil and root samples of pistachio trees were surveyed. Totally, 78 soil and root samples were collected from pistachio orchards in Ardakan city of Yazd Province. Nematodes were extracted from roots and the rhizosphere soil using the tray method (Whitehead and Hemming, 1965) and after 48h the nematodes at the bottom of tray were washed with tap water and kept for both morphological and molecular analysis. In morphological studies the nematodes fixed in TAF (Triethanolamine 2  ml, formaldehyde 7  ml, distilled water 91ml), and transferred to glycerin (De Grisse, 1969). Primary identification was carried out on the basis of morphometric plus morphological characters of adult females (Gafur, 2020). Specimens were examined using an Olympus compound microscope at powers up to 100 × magnification. Measurements and drawings were made by drawing tube on glycerin infiltrated specimens. All measurements were expressed in micrometer (μm). All other abbreviations used are as defined in Siddiqi (2000).

### DNA extraction, amplification, sequencing and phylogenetic analysis

For molecular analyses, DNA extraction were achieved based on the method of Tanha Maafi et al. (2003). About 10 adult females were put in 8 μl ddH_2_O on a glass slide and punctured under a dissecting microscope. Adult females were transferred to an Eppendorf tube containing 12  μl worm lysis buffer (500 mM KCl, 100 mM Tris-Cl pH 8, 15 mM MgCl_2_, 10 mM DTT, 4.5% Tween 20) and crushed with a microhomogeniser Vibro Mixer (Zürich, Switzerland). Two microliters proteinase K (600 μg/ml) (Promega Benelux, Leiden, The Netherlands) were added and the tubes were frozen at ‒80°C for at least 10  min and then incubated at 65°C (1 hr) and 95°C (10  min) consecutively. After incubation, the tubes were centrifuged for 2  min at 14,000  rpm and kept at ‒20°C until use. The PCR was carried out in a 30  μl reaction comprising of 2  µL DNA template, 1  μl forward and reverse primers, 15  μl Taq DNA Polymerase 2 × Master Mix (Ampliqon), and 11  μl distilled water. The forward primer D2A (5′-ACAAGTACCGTGA GGGAAAGTTG-3′) and the reverse primer D3B (5′-TCGGAAGGAACCAGCTACTA-3′) (Subbotin et al., 2006) were used for amplification of the D2–D3 expansion region of the 28S rRNA gene. The PCR amplification profile consisted of 4 min at 95°C; 33 cycles of 30 sec at 95°C, 40 sec at 53°C and 30 sec at 72°C, followed by a final step of 7  min at 72°C. The PCR products were run on a, 1.0% agarose gel in 1 × TBE buffer stained and photographed. PCR product was purified and sequenced in both directions (Bioneer Company, South Korea) then newly obtained sequence was deposited in GenBank database under accession number MW338666 and was compared with sequences previously deposited in GenBank (NCBI). The species-specific PCR reaction was performed with *P. oleae*-specific primer pairs Poleae_fw1_4_36 (5′-GACAGATTAGAATGGAATCTGTTCG-3′) and Poleae_rv1_ _525_551 (5′ATCGCTTTTGGATTCAATAATATA-3′) as described by Palomares-Rius et al. (2014). For phylogenetic analysis, obtained *P. oleae* sequence was aligned with related sequences from GenBank through National Center for Biotechnology Information (NCBI) BLASTn homology search, using ClustalW implemented by MEGA version 10.0. The GTR + I + G model was selected as the best by jModeltest v.2.1.10. Bayesian tree generated using the Bayesian inference method as implemented in the program MrBayes v.3.2.6 (Huelsenbeck and Ronquist, 2001). Markov Chain Monte Carlo (MCMC) methods ran chains for 1,000,000 generations and setting the ‘burnin’ at 2,500 (l set number of substitution types = 6, rates = invgamma, number of rate categories for the gamma distribution = 4, sampling frequency = 100 generations). The *Coslenchus costatus*, *Boleodorus* sp. and *Basiria gracilis* were selected as outgroups for datasets (Majd Taheri et al., 2013).

## Results

**Figure 1:**
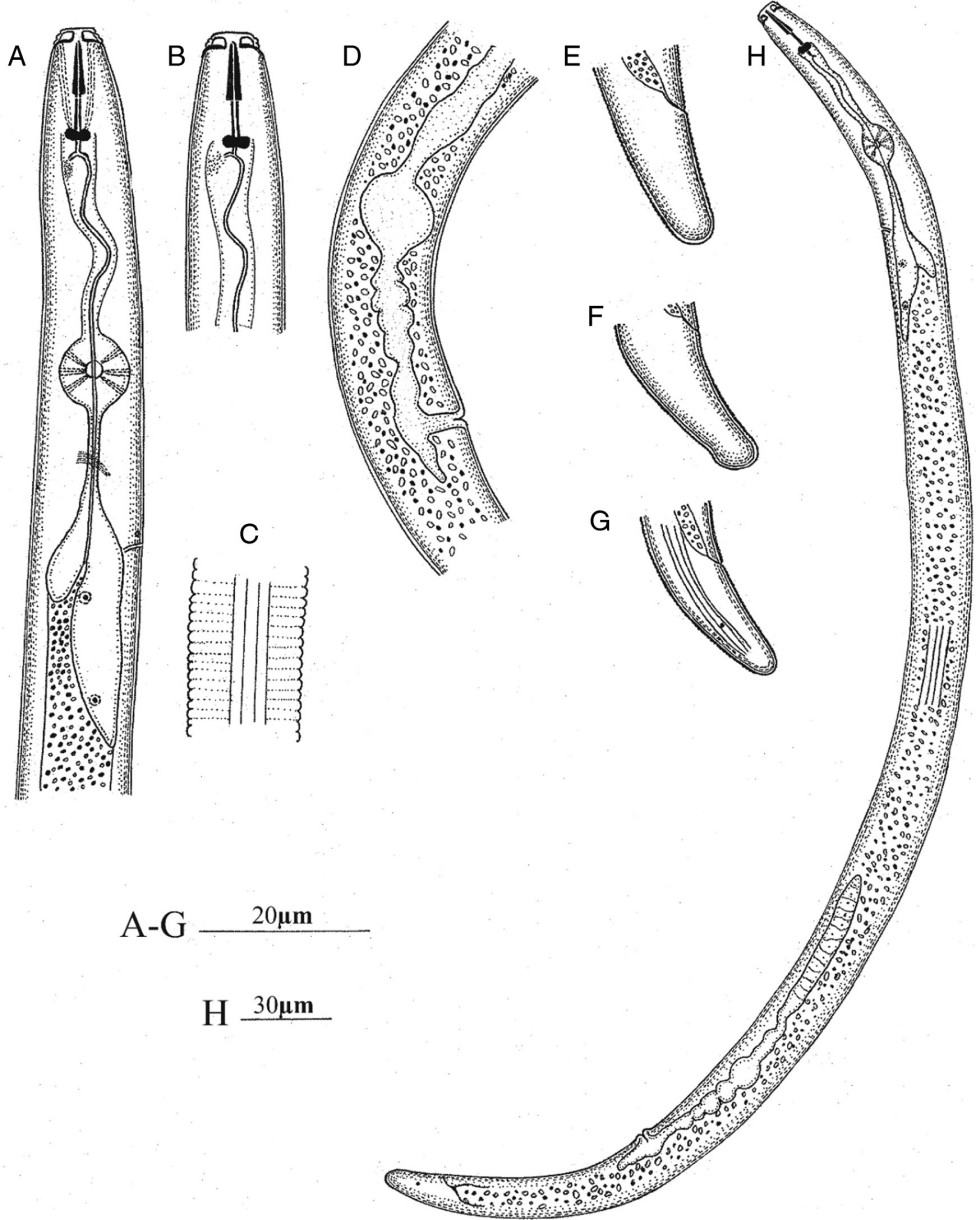
Iranian isolate of *Pratylenchus oleae*. A and B: Anterior region; C: Lateral field at mid-body; D: Genital tract and vulva region; E-G: Variation in tail shape, (H) Adult female.

Morphometric of *P. oleae*, found in roots and around soil from Pistachio tree in Iran are presented in Table 1. The morphological and molecular analyses confirmed that the species was *P. oleae* as well. *P. oleae* is characterized by morphological features in females: Vermiform body, lip region with three annuli, slightly offset. Stylet well developed with distinct rounded knobs slightly directed anteriorly. Lateral field with four incisures, metacorpus oval to rounded, isthmus rather short, surrounded by nerve ring, pharyngeal glands well developed, with rather long ventral overlap. Excretory pore either slightly anterior pharyngo-intestinal junction or opposite. Genital branch with one row of oocyte, spermathecal rounded, vagina a straight tube. Vulva transverse slit. Post-vulval uterine sac short, not differentiated. Tail sub-cylindrical, terminus rounded to conical, smooth, male unknown (Fig. 1). *P. oleae* is morphologically closely related to *P. cruciferus*, *P. delattrei* and *P. kumamotoensis.*

**Table 1.** Morphometric characters of Iranian isolate of *Pratylenchus oleae* on different isolates.

Character	Yazd – Ardakan (Iran)	Agua Amarga-Níjar (Spain)	Albaricoques-Níjar (Spain)	Ouled Chamekh (Tunisia)
n	10	20	8	14
L	522.5 ± 34.9 (463.0–565.0)	455 ± 30.4 (412–511)	464 ± 31.6 (416–513)	501 ± 37.8 (440–555)
a	28.3 ± 1.6 (25.7–31.0)	24.4 ± 1.8 (21.0–27.8)	25.1 ± 1.8 (22.5–27.0)	28.1 ± 1.4 (26.9–32.6)
b	5.9 ± 0.4 (5.2–6.7)	4.5 ± 0.3 (4.0–5.4)	4.6 ± 0.3 (4.0–5.2)	5.0 ± 0.6 (4.3–5.9)
b´	4.2 ± 0.3 (3.6–4.7)	-	-	-
c	20.2 ± 0.8 (19.1–21.0)	20.2 ± 1.8 (17.0–24.3)	19.1 ± 1.0 (17.0–20.1)	22.1 ± 2.1 (19.0–25.2)
c´	2.1 ± 0.1 (1.8–2.3)	2.0 ± 0.2 (1.7–2.4)	2.2 ± 0.1 (2.0–2.4)	1.9 ± 0.3 (1.7–2.3)
V	80.8 ± 1.5 (77.8–83.0)	80.1 ± 1.3 (78.0–82.0)	80.2 ± 1.3 (78.0–82.0)	79.3 ± 1.5 (78.5–81.5)
Stylet Length	17.3 ± 0.7 (16.0–18.0)	16.5 ± 0.6 (14.5–17.0)	16.4 ± 0.7 (15.0–17.0)	15.8 ± 0.6 (15.0–17.0)
Median Bulb	55.6 ± 2.3 (51.0–59.0)	46.4 ± 1.7 (44.0–51.0)	46.3 ± 1.6 (44.9–49.6)	47.6 ± 3.0 (42.0–50.9)
Anterior to Excretory Pore	88.9 ± 2.7 (85.0–93.0)	82.0 ± 5.3 (71.0–95.0)	82.4 ± 5.8 (71.0–91.0)	78.0 ± 5.2 (73.0–87.0)
Anterior End to Pharyngeal Junction	89.0 ± 4.6 (78.0–94.0)	-	-	-
Pharynx Length	123.5 ± 3.4 (118.0–129.0)	102.0 ± 7.6 (90.0–113.0)	100.1 ± 4.3 (92.0–106.0)	96.8 ± 7.1 (89.0–110.0)
Max. Body Diam.	18.5 ± 1.0 (17.0–20.0)	-	-	-
Body Diam. at Anus	12.6 ± 0.5 (12.0–13.0)	-	-	-
Tail Length	25.9 ± 1.7 (22.0–27.0)	22.7 ± 2.2 (19.0–26.5)	24.4 ± 1.7 (21.0–27.0)	23.1 ± 3.1 (19.0–29.0)

Morphometric mean, standard deviation and range values of *P. oleae* females were (*n*  =  10): *L*  =  522.5 ± 34.9 (463.0–565.0) μm; *a*  =  28.3 ± 1.6 (25.7–31.0)  μm; *b*  =  5.9 ± 0.4 (5.2–6.7)  μm; *c*  =  20.2 ± 0.8 (19.1–21.0) μm; Stylet length  =  17.3 ± 0.7 (16.0–18.0) μm; Median bulb 55.6 ± 2.3 (51.0–59.0) μm; Anterior to Excretory Pore 88.9 ± 2.7 (85.0–93.0) μm; Anterior end to pharyngeal junction 89.0 ± 4.6 (78.0–94.0) μm; Pharynx Length 123.5 ± 3.4 (118.0–129.0) μm; Max. Body diam. 18.5 ± 1.0 (17.0–20.0) μm; Body diam. at anus 12.6 ± 0.5 (12.0–13.0)  μm and tail length  =  25.9 ± 1.7  (22.0–27.0)  μm (Table 1).

**Figure 2:**
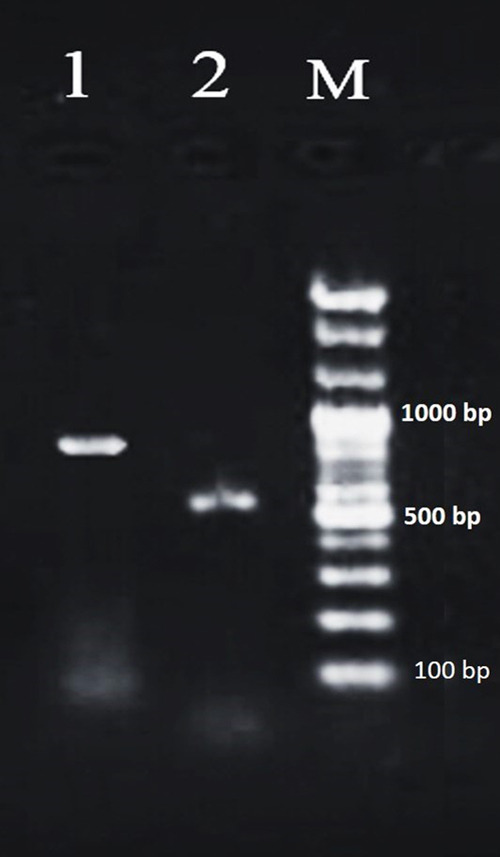
The gel with PCR amplicons obtained with D2–D3 expansion segments of 28S rRNA sequences (Line 1) and species specific primers for *Pratylenchus oleae* (Line 2). M – 100 bp DNA Marker.

The D2–D3 region of the 28S-rDNA amplified with the primer sets D2A/D3B and yielded single fragments of 800 bp (Fig. 2), based on direct fragment sequencing. The blastn test of 28S rDNA showed that only seven accession numbers of *P. oleae* belong to Spain (three isolates of Agua Amarga-Nijar, Almeria, wild olive and one isolates of Albaricoques-Nijar, Almeria, cultivated olive Picnal) and Tunisia (three isolates of Ouled Chamekh, cultivated olive Koroneiki). *Pratylenchus capsici*, 9 isolates are most similar to *P. oleae*, but differs in presence of males, a functional spermatheca, a larger body and shorter stylet (Qing et al., 2019). BLAST searches indicated 99–100% identity (similarity) with sequences of *P. oleae* and 100% similarity with sequences of *P. capsic*i (Table 2). Sequences from other species of *Pratylenchus* obtained from NCBI were used for further phylogenetic studies. D2–D3 expansion segments of 28S rRNA of *P. oleae* were deposited in GenBank under the accession numbers MW338666 from cultivated pistachio trees cv. Badami from Ardakan city, Yazd province matched well with the *Pratylenchus* spp. deposited in GenBank. This sequence had intra-specific variation 100% similarity (2 nucleotide differences) with *P. oleae* Spain isolate (KJ510861) and 99% (2–7 nucleotide differences) similar to *P. oleae* Spain isolates (KJ510855, KJ510856, KJ510857) and *P. oleae* Tunisia isolates (KJ510858, KJ510859, KJ510860), while *P. dunensis* and *P. penetrans* are 89% and 87% similar (61 and 82 nucleotide differences with 6 and 13 Gaps). The gap variations and nucleotide differences between 9 isolates of *P. capsici* and *P. oleae* (MW338666) are between 3–9 and 30–37 nucleotides respectively (Table 2).

**Table 2.** Phylogenetic characters of Iranian isolate of *Pratylenchus oleae* (MW338666) compared to others isolates.

Species	Genebank Accession/28S-rDNA	Similarity	Query Cover	Nucleotide Differences	Gap
*Pratylenchus oleae*	KJ510855	99%	79%	2	0
*Pratylenchus oleae*	KJ510856	99%	100%	7	1
*Pratylenchus oleae*	KJ510857	99%	100%	7	1
*Pratylenchus oleae*	KJ510858	99%	98%	5	1
*Pratylenchus oleae*	KJ510859	99%	100%	7	2
*Pratylenchus oleae*	KJ510860	99%	84%	6	2
*Pratylenchus oleae*	KJ510861	100%	79%	2	0
*Pratylenchus capsici*	MH796977	100%	94%	31	3
*Pratylenchus capsici*	MH796976	100%	94%	32	3
*Pratylenchus capsici*	MH796970	100%	93%	37	4
*Pratylenchus capsici*	MH796975	100%	95%	31	4
*Pratylenchus capsici*	MH796978	100%	94%	33	4
*Pratylenchus capsici*	MH796974	100%	93%	34	9
*Pratylenchus capsici*	MH796969	100%	93%	32	9
*Pratylenchus capsici*	MH796971	100%	93%	30	6
*Pratylenchus capsici*	MH796973	100%	94%	33	6
*Pratylenchus dunensis*	AJ890459	89%	99%	61	6
*Pratylenchus penetrans*	JX261961	87%	100%	82	13

**Figure 3:**
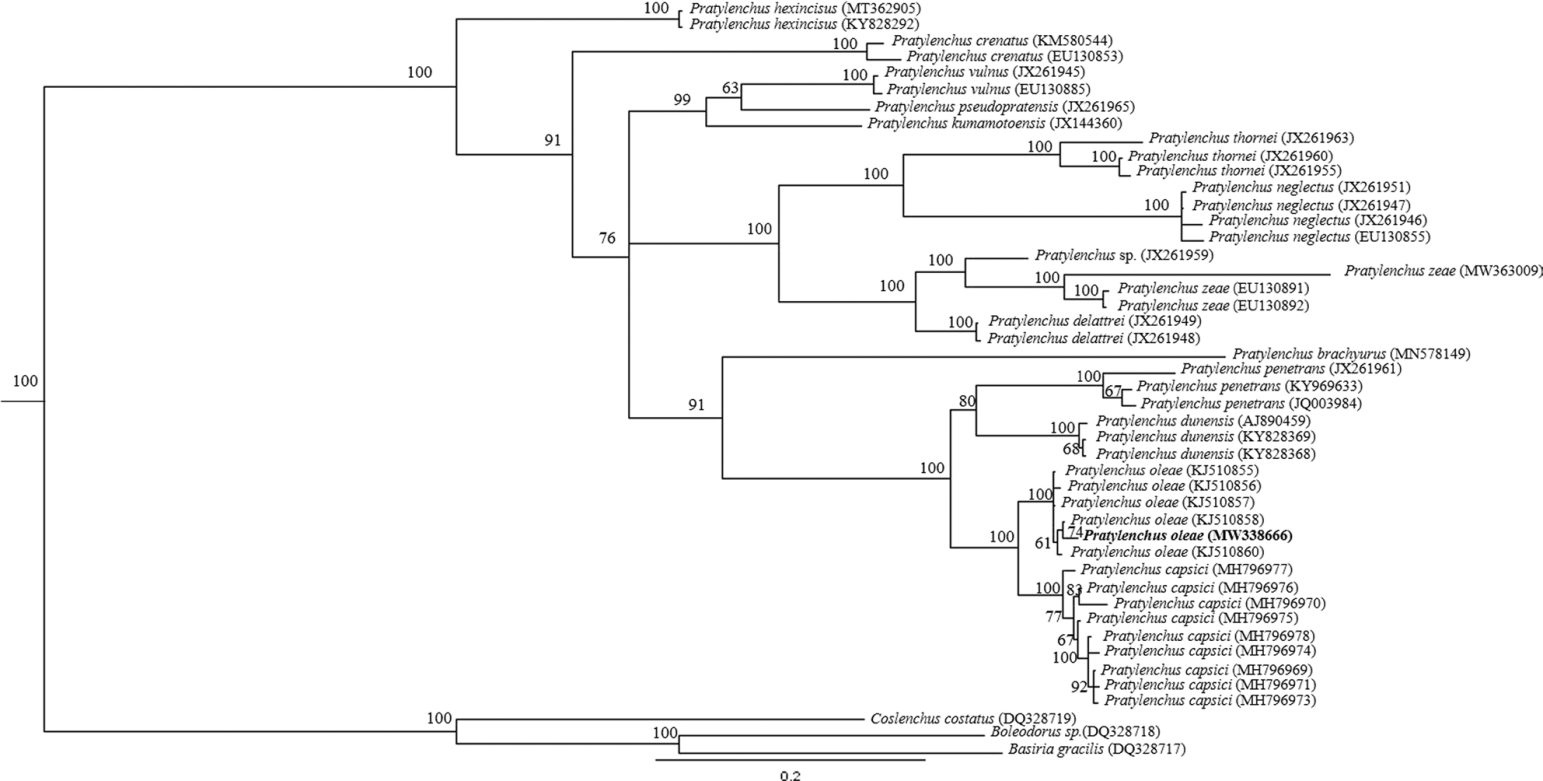
The Bayesian tree inferred from known and newly sequenced *Pratylenchus oleae* from IRAN based on the 28S rDNA region under GTR + I + G model.

In total, 46 sequences were included in the phylogenetic analysis of D2-D3 expansion segments of the 28S rDNA gene. The phylogenetic tree based on 28S rDNA, placed the Iran *P. oleae* population in a clade together with other *P. oleae* populations and revealed the strong relationship of *P. oleae* with *P. penetrans, P. dunensis* and *P. capsici*. (Fig. 3). The molecular characterization of several isolates of *P. oleae* suggested that they formed a monophyletic group. To the best of our knowledge, this is the first record of *P. oleae* from pistachio rhizosphere and root in the world. The species identification of *P. oleae* was confirmed using PCR by species-specific primers Poleae_fw1_4_36/Poleae_rv1_ _525_551 and produced a 547-bp fragment (Fig. 2), which was the same as reported by Palomares-Rius et al. (2014).

## Discussion

Their morphology and morphometric of *P. oleae* population in Iran followed the original species descriptions, which were reported previously from two locations of Spain (Agua Amarga-Níjar and Albaricoques-Níjar) and one location of Tunisia (Ouled Chamekh) (Palomares-Rius et al., 2014), But some morphometric discrepancies were found. Body, stylet length, anterior to excretory pore, pharynx length, tail length of Iranian specimen was slightly 68 μm, 0.8 μm, 6.9 μm, 21.5 μm and 3.2 μm longer, respectively. The Iranian isolate of *P. oleae* is characterized by lip region consisting of three annuli and four incisures along with oblique lines in middle of body, tail sub-cylindrical to mild conoid like to other *P. oleae* isolates.

The sequence of Iranian isolate showed high nucleotide similarity with D2-D3 region of *P. oleae* deposited in GenBank database. The test population had no gap (base pair), which was different to those of *P. oleae* from Spain (KJ510855 and KJ510861) with 100% similarity, one gap with Spain (KJ510856 and KJ510857) and Tunisia (KJ510858) with 99% similarity respectively and two gaps with Tunisia (KJ510859 and KJ510860) with 99% similarity. In summary, Iranian *P. oleae* was isolated from pistachio trees in Ardakan, Yazd province. It is reported for the first time from Iran and pistachio tree in the world. This nematode can be considered as a risk to the economy in pistachio orchards.
